# Association of daily health behavior and activity of daily living in older adults in China

**DOI:** 10.1038/s41598-023-44898-7

**Published:** 2023-11-09

**Authors:** Ling-Ying Wang, Mei Feng, Xiu-Ying Hu, Meng-Lin Tang

**Affiliations:** 1 West China Hospital Critical Care Medicine Department, Sichuan University/West China School of Nursing, Sichuan University, Chengdu, 610041 China; 2 Innovation Center of Nursing Research and Nursing Key Laboratory of Sichuan Province, West China Hospital, Sichuan University/West China School of Nursing, Sichuan University, Chengdu, 610041 China

**Keywords:** Health care, Risk factors

## Abstract

This study aims to describe the activity of daily living (ADL) situation and determine the relationship between health behavior and ADL among older adults in China. A cross-sectional, observational study was conducted in one urban community and one rural community in Chengdu (a city located in Southwest China), China, from October 2022 to March 2023. A total of 706 older adults were included in this study. The associations between health behaviour and ADL were assessed by logistic regression model. Of the 706 older adults, 169 (23.9%) were disabled in ADL. According to the logistic regression analysis, age (60–69 years old: OR = 0.015, 95% CI 0.007 to 0.035, *P* < 0.001; 70–79 years old: OR = 0.116, 95% CI 0.060 to 0.227, *P* < 0.001), resident(OR = 0.568, 95% CI 0.330 to 0.976, *P* = 0.041), chronic disease (0 type: OR = 0.023, 95% CI 0.001 to 0.379, *P* = 0.008; 1–4 types: OR = 0.357, 95% CI 0.219 to 0.582, *P* < 0.001), no exercise (OR = 4.562, 95% CI 2.263 to 8.026, *P* < 0.001), and physical examination (OR = 2.217, 95% CI 1.294 to 3.496, *P* = 0.003) were significantly associated with ADL among older adults in Southwest China. This study showed that older adults had a higher ADL disability ratio. Age, resident, chronic disease, exercise and physical examination were associated with ADL among older adults. The study indicates that medium/high exercise maybe a protective factor for older adults, and nursing staff can encourage older adults to exercise when carrying out primary prevention measures. The government and public health institutions should give special attention to older adults and help them to acquire the habit of having an annual physical examination.

## Introduction

People continue to live longer and live more years in good health. Global life expectancy at birth increased from 66.8 years in 2000 to 73.3 years in 2019, and healthy life expectancy (HALE) increased from 58.3 years to 63.7 years. The gap between life expectancy and health expectancy has remained at approximately 10 years, and reduction of this gap has been of concern^1^. To achieve this goal, a possible approach is to eliminate risk factors that contribute to becoming dependent on others for basic activities of daily living (ADL). In general, demographic characteristics, lifestyle habits, and physical health status were associated with older adults' daily living activity ability, including female gender, advanced age, lower education, habitual smoking, restricted daily activity, being bedridden, higher pain levels and so on^2,3^.

Health behavior as an intervention behavior that can be led by nursing staff is significant in improving the health of elderly individuals. Health behavior is a positive action by the elderly to prevent disease and maintain health; it includes changing dangerous lifestyles, reducing or eliminating health-risk behavior (e.g., smoking, alcohol, and unhealthy diet), taking active health behavior (e.g., regular exercise and regular physical examination), and complying with doctors' recommendations. It is one of the most effective approaches to control noncommunicable diseases and save medical expenses in the elderly^4^. A study has shown that nurse-led health coaching may lead to an improvement in health-related quality of life^5^.

Several studies reported that health literacy was significantly associated with health-related behaviors in elderly individuals^4,6,7^, as they only focused on smoking, alcohol consumption, regular exercise, and dietary behavior. Sun et al.^8^ found that sleep duration was positively correlated with the risk of ADL impairment in elderly individuals in a nonlinear dose‒response relationship. Tański et al.^9^ found that sleep disorders correlate negatively with quality of life scores in the physical and mental domains. Hou’s^10^ study reported that health awareness, such as participating in regular physical examinations, had a significant influence on ADL in elderly individuals.

To our knowledge, no prior study has estimated the association between daily health behaviors, including tobacco use, alcohol use, exercise, dietary behavior, sleep behavior and physical examination, and ADL among older adults in China over the past decade. The purpose of this study was to determine the relationship between health behavior and ADL among elderly individuals in China.

## Methods

### Study population and recruitment

A cross-sectional, observational study was conducted in one urban community and one rural community in Chengdu (a city located in Southwest China), China, from October 2022 to March 2023. During this time, all older adults who met the following criteria were included in this study: (i) age ≥ 60 years and (ii) agreed to cooperate with the investigators after the purpose of the research was explained. Exclusion criteria: (i) Mental disorders, severe, and end-stage diseases; (ii) The respondents had ADL impairment due to acute illness/accident.

The research team collected data after obtaining their consent and signatures on the study's informed consent form. The study was approved by the Ethics Committee of West China Hospital, Sichuan University in 2022 (Ethics No. 861). All methods were performed in accordance with the relevant guidelines and regulations. Informed consent was obtained from all participants.

### Procedure and data collection

The research team was composed of research nurses and the first author. In our study, the main exposure variables including health behaviors.

#### Sociodemographic variables

We designed a questionnaire to collect sociodemographic data from the participants. The variables included age, sex, height, weight, marital status, nationality, educational level, and current chronic illnesses.

We calculated BMI by the equation below: BMI = weight (in kg)/height^2^ (in m^2^). An individual was divided into severely underweight (BMI less than 16.5 kg/m^2^), underweight (BMI under 18.5 kg/m^2^), normal weight (BMI greater than or equal to 18.5 to 23 kg/m^2^), overweight (BMI between 23 and 24.9 kg/m^2^), and obese (BMI greater than 25 kg/m^2^) groups^11^.

#### Activity of daily living (ADL)

We used the Barthel Index (BI), designed by Mahoney^12^ and Barthel in 1965, to evaluate basic activities of daily living (ADL), including feeding, bathing, grooming, dressing, bowels and bladder, toilet use, transfers, mobility, and climbing stairs. Individual scores of the ten items range from 0 (total dependence) to 100 (complete independence). Its use has spread rapidly due to its simplicity, and it has been modified various times^13–15^. The BI survey was offered in Chinese, translated and introduced by Hou^16^. We divided the individuals into complete independence (BI scores = 100) and dependence (BI scores < 100) groups^17,18^.

#### Daily health behaviors

We assessed 6 health behaviors that are important daily lifestyle features that require improvement: tobacco use, alcohol use, exercise, dietary behavior, sleep behavior and physical examination.

- (1) Tobacco use

Tobacco use was divided into current, former and never smokers.

- (2) Alcohol use

Alcohol use was divided into current, former and never drinkers.

- (3) Exercise

We used the physical activity rating scale (PARS-3) designed by Hashimoto^19^ and translated and revised by Liang et al.^20^, which is a 3-item self-reported scale comprising intensity, duration and frequency. Participants were asked to rate the intensity, duration and frequency of their bodily movements from 1 to 5, and the total score of physical activity (i.e., exercise volume) was computed by the equation below: intensity × (duration − 1) × frequency, ranging from 0 to 100. Physical activity is divided into three levels, including light exercise (≤ 19), medium exercise (20–42), and high exercise (≥ 43)^21^. Furthermore, in accordance with previous experience^22,23^, our study divided light exercise into two levels: none exercise (≤ 4) and low exercise (5–19).

- (4) Dietary behaviour

Dietary behavior included regular eating patterns, balanced meals, and dietary quality. Regular eating patterns included meal time and frequency. We used the Food Frequency Questionnaire (FFQ)^24^ to evaluate balanced meals, based on the Dietary Guidelines for Chinese Residents and the eating habits of Southwest people, consisting of questions about thirteen main food groups, including staple foods, beans, livestock, poultry, fish, eggs, dairy, fruits, vegetables, wine nuts, pickles, and beverages. Participants reported their usual consumption frequency over the past year, according to eight categories: never, 1–3 times/month, 1–2 times/week, 3–4 times/week, 5–6 times/week, 1 time/day, 2 times/day and ≥ 3 times/day^25^. The consumption of household seasonings, such as cooking oil, salt, sugar, sauce, etc., is determined by evaluating all seasonings consumed by all family members within a month. The total consumption of condiments in a family divided by the number of members who usually eat at home is used to evaluate the consumption of personal condiments^26^.

The dietary quality among the participants was evaluated using the Chinese Diet Balance Index 2016 (DBI-16)^27^, which includes 14 subgroups of 8 components from the Dietary Guidelines for Chinese residents^28^, including (1) cereal; (2) vegetable and fruit; (3) dairy and soybean; (4) animal food (red meats/products/poultry/game, fish/shrimp, and eggs); (5) empty energy foods (cooking oils and alcoholic beverages); (6) condiments (addible sugar and salt); (7) diet variety; and (8) drinking water. A score of 0 for each DBI-16 component means that the individual has reached the recommended intake amounts of the corresponding food group. Positive scores (ranging from 1 to 12) indicate an excessive intake level of cereals, red meat/products/poultry/game, eggs, cooking oils, alcoholic beverages, addible sugar, and salt, while negative scores (ranging from − 12 to − 1) indicate inadequate intake levels of cereals, vegetables, fruits, dairy, soybeans, red meat/products/poultry/game, fish/shrimps, eggs, diet variety, and drinking water. Considering the differences in nutritional requirements for energy consumption, the scoring of these 14 food subgroups was based on 11 energy intake levels.

Based on the scores for each DBI-16 component, three dietary quality indicators were calculated: (1) the lower bound score (LBS), an indicator for insufficient food intake, calculated by adding all the negative scores; (2) the higher bound score (HBS), an indicator for excessive food intake, calculated by adding all the positive scores; and (3) the diet quality distance (DQD), an indicator of unbalanced food intake, calculated by adding the absolute values of both positive and negative scores^27^. The ranges of LBS, HBS, and DQD are 0 to 60, 0 to 40, and 0 to 84, respectively. Each indicator is further divided into five levels to reflect diet quality: (1) no problem, a score of 0; (2) almost no problem, less than 20% of the total score; (3) low-level problem, between 20 and 40% of the total score; (4) moderate-level problem, between 40 and 60% of the total score; and (5) high-level problem, greater than 60% of the total score.

## (5) Sleep behavior

In our study, sleep behavior included afternoon nap habits and sleep quality. Sleep quality was evaluated using the global Pittsburgh Sleep Quality Index (PSQI) Score^29^. The questionnaire consists of 18 items under 7 main components, including (i) subjective sleep quality, (ii) sleep latency, (iii) sleep duration, (iv) habitual sleep efficiency, (v) sleep disturbances, (vi) use of sleep-promoting medications, and (vii) daytime dysfunction, based on the past month. Each component of the questionnaire is scored from 0 to 3 (representing good to bad), resulting in a global PSQI score ranging from 0 to 21, with higher scores indicating worse sleep quality^29^. A global PSQI score greater than 5 has been found to have a sensitivity of 89.6% and specificity of 86.5% in differentiating good sleepers from poor sleepers^30^. The PSQI survey was offered in Chinese, translated and introduced by Liu, and the Cronbach’s α coefficient was 0.842^31^.

## (6) Physical examination

Physical examination was assessed by whether the individual had regular physical examination (≥ 1 time/year).

Before the initiation of the formal investigation, the first author trained and educated 2 nurses in subjects on how to complete the questionnaire. There was an assessment at the end of the training, and the 2 nurses who were employed as the research nurses in the study had similar assessment scores, ages, years of experience, levels of education, and job titles. Finally, these 2 nurses were able to complete the questionnaire with unified standards before beginning the formal investigation.

### Statistical analysis

The whole project was a prospective study and this paper was a pre-planned study which analyzed baseline data. All data were categorical variables. Descriptive statistics were presented by counts and percentages to describe the demographic information. The counts and percentages were entered into the Statistical Package for the Social Sciences (version 24.0). The chi-squared test was used to assess the association of demographic and daily healthy behavior with ADL. Statistical significance was set at *P* < 0.05 (two-tailed). We merged adjacent groups with similar clinical significance, with a sample size of less than 5% within the group.

All general data and daily healthy behavior data of the individuals were considered independent variables and put into multivariate analysis, and ADL decline was considered the dependent variable in the analysis. The B, SE (standard error), Wald, 95% CI, and P values were reported. The results of the model were reported as ORs (95% CIs).

### Patient and public involvement

Former individuals were involved in the preparatory phase of this study. They reviewed the informed consent form and provided feedback.

## Results

During the study, 706 older adults were observed. The general demographic data and ADL are reported in Table 1. Among them, 48.4% of the older adults were 60–69 years old, 57.1% of the older adults were male, 56.5% of the older adults lived in urban districts, and 96.2% of the older adults were Han nation. A total of 436 older adults had a low education, 88.5% of them were married, and 36.3% of them had normal weight. A total of 26.1% of the older adults had ≥ 5 chronic diseases, and 23.9% of them were disabled in ADL.

**Table 1:** Characteristics of the older adults observed in this study.

Variable	n	%
Age (years)		
60–69	342	48.4
70–79	264	37.4
≥ 80	100	14.2
Sex		
Male	403	57.1
Female	303	42.9
Resident		
Urban	399	56.5
Rural	307	43.5
Nation		
Han	679	96.2
Minority groups	27	3.8
Education level		
Low	436	61.8
Middle	140	19.8
High	130	18.4
Marriage		
Married	625	88.5
Non-married	81	11.5
BMI		
Severely underweight/Underweight	32	4.5
Normal weight	256	36.3
Overweight	180	25.5
Obesity	238	33.7
Chronic disease (types)		
0	36	5.1
1–4	486	68.8
≥ 5	184	26.1
ADL		
Self-care	537	76.1
Disabled	169	23.9

Table 2 shows a comparison of demographic and daily health behaviors with significant associations in univariate analyses that contributed to ADL in Southwest China. Our results showed that there were significant differences in ADL among different ages (*P* < 0.001); 4.7% of the older adults were disabled in the 60–69 years old age group, 30.3% were disabled in the 70–79 years old age group, and 73.0% were disabled in the group (age ≥ 80 years old). In addition, we found that there were significant differences in ADL among sex (*P* < 0.001), education level (*P* = 0.046), marriage (*P* < 0.001), chronic disease (*P* < 0.001), exercise (*P* < 0.001), number of minutes to fall asleep (*P* < 0.001), sleep quality (*P* = 0.002), physical examination (*P* < 0.001), HBS (*P* = 0.041), LBS (*P* = 0.007), tobacco use (*P* = 0.009) and alcohol use (*P* = 0.003) factors.

**Table 2:** Results of univariate analyses of associations between demographic and daily healthy behavior factors and ADL in the southwest of China.

Variable	ADL		χ^2^	*P* value
	Self-care	Disabled		
Age (years)				
60–69	326 (95.3)	16 (4.7)	207.751	< 0.001
70–79	184 (69.7)	80 (30.3)		
≥ 80	27 (27.0)	73 (73.0)		
Sex				
Male	329 (81.6)	74 (18.4)	16.031	< 0.001
Female	208 (68.6)	95 (31.4)		
Resident				
Urban	313 (78.4)	86 (21.6)	2.864	0.091
Rural	224 (73.0)	83 (27.0)		
Nation				
Han	515 (75.8)	164 (24.2)	0.453	0.501
Minority groups	22 (81.5)	5 (18.5)		
Education level				
Low	318 (72.9)	118 (27.1)	6.146	0.046
Middle	113 (80.7)	27 (19.3)		
High	106 (81.5)	24 (18.5)		
Marriage				
Married	490 (78.4)	135 (21.6)	16.350	< 0.001
Non-married	47 (58.0)	34 (42.0)		
BMI				
Severely underweight/Underweight	23 (71.9)	9 (28.1)	4.685	0.196
Normal weight	184 (71.9)	72 (28.1)		
Overweight	142 (78.9)	38 (21.1)		
Obesity	188 (79.0)	50 (21.0)		
Chronic disease (types)				
0	35 (97.2)	1 (2.8)	65.245	< 0.001
1–4	401 (82.5)	85 (17.5)		
≥ 5	101 (54.9)	83 (45.1)		
Exercise				
None exercise	58 (47.5)	64 (52.5)	87.500	< 0.001
Low exercise	179 (73.1)	66 (26.9)		
Medium/High exercise	300 (88.5)	39 (11.5)		
PSQI				
Good sleepers	347 (80.0)	87 (20.0)	9.370	0.002
Poor sleepers	190 (69.9)	82 (30.1)		
Physical examination				
None	155 (67.1)	76 (32.9)	15.148	< 0.001
Yes	382 (80.4)	93 (19.6)		
Eating patterns				
< 3 meals/day	18 (69.2)	8 (30.8)	1.591	0.451
3 meals/day	511 (76.2)	160 (23.8)		
> 3 meals/day	8 (88.9)	1 (11.1)		
HBS				
No problem	12 (54.5)	10 (45.5)	5.741	0.041
Almost no problem	513 (76.6)	157 (23.4)		
Low-level problem	12 (85.7)	2 (14.3)		
LBS				
Almost no problem	398 (79.3%)	104 (20.7)	9.897	0.007
Low-level problem	122 (68.2)	57 (31.8)		
Moderate level problem	17 (68.0)	8 (32.0)		
DQD				
Almost no problem	416 (77.5)	121 (22.5)	2.836	0.242
Low-level problem	116 (71.2)	47 (28.8)		
Moderate level problem	5 (83.3)	1 (16.7)		
Tobacco use				
Never smokers	345 (72.6)	130 (27.4)	9.413	0.009
Former smokers	115 (82.7)	24 (17.3)		
Current smokers	77 (83.7)	15 (16.3)		
Alcohol use				
Never drinkers	375 (72.8)	140 (27.2)	11.571	0.003
Former drinkers	75 (82.4)	16 (17.6)		
Current drinkers	87 (87.0)	13 (13.0)		

*PSQI* pittsburgh sleep quality index; *LBS* the lower bound score; *HBS* the higher bound score; *DQD* the diet quality distance.

The final logistic regression analysis in Table 3 showed that age (60–69 years old: OR = 0.015, 95% CI 0.007 to 0.035, *P* < 0.001; 70–79 years old: OR = 0.116, 95% CI 0.060 to 0.227, *P* < 0.001), resident (OR = 0.568, 95% CI 0.330 to 0.976, *P* = 0.041), chronic disease (0 type: OR = 0.023, 95% CI 0.001 to 0.379, *P* = 0.008; 1–4 types: OR = 0.357, 95% CI 0.219 to 0.582, *P* < 0.001), no exercise (OR = 4.562, 95% CI 2.263 to 8.026, *P* < 0.001), and physical examination (OR = 2.217, 95% CI 1.294 to 3.496, *P* = 0.003) were significantly correlated with ADL among older adults in Southwest China.

**Table 3:** Logistic regression model of risk factors for ADL among older adults in the southwest of China.

Variable	B	S.E	Wald	OR	95% CI		*P* Value
					Lower	Upper	
Age (years)			97.288				** < 0.001**
60–69	− 4.181	0.424	97.229	0.015	0.007	0.035	** < 0.001**
70–79	− 2.151	0.341	39.875	0.116	0.060	0.227	** < 0.001**
Sex (male)	− 0.588	0.320	3.370	0.555	0.296	1.041	0.066
Resident (urban)	− 0.566	0.276	4.194	0.568	0.330	0.976	**0.041**
Nation (Han)	0.400	0.710	0.317	1.491	0.371	5.995	0.573
Education level			0.731				0.694
Low	0.313	0.411	0.582	1.368	0.612	3.058	0.446
Middle	0.114	0.460	0.061	1.120	0.455	2.759	0.805
Marriage (married)	− 0.036	0.355	0.011	0.965	0.501	1.859	0.915
BMI			2.392				0.495
Severely underweight /Underweight	− 0.733	0.595	1.518	0.480	0.150	1.542	0.218
Normal weight	− 0.361	0.299	1.462	0.697	0.388	1.251	0.227
Overweight	− 0.307	0.323	0.902	0.736	0.390	1.386	0.342
Chronic disease (types)			21.766				** < 0.001**
0	− 3.760	1.423	6.984	0.023	0.001	0.379	**0.008**
1–4	− 1.029	0.249	17.105	0.357	0.219	0.582	** < 0.001**
Exercise			20.369				** < 0.001**
None exercise	1.450	0.323	20.145	4.262	2.263	8.026	** < 0.001**
Low exercise	0.467	0.278	2.816	1.595	0.925	2.752	0.093
PSQI (good sleepers)	− 0.008	0.249	0.001	0.992	0.609	1.615	0.974
Physical examination (none)	0.755	0.254	8.855	2.127	1.294	3.496	**0.003**
Eating patterns			0.658				0.720
< 3 meals/day	− 0.126	0.653	0.037	0.881	0.245	3.168	0.847
3 meals/day	− 1.195	1.516	0.621	0.303	0.016	5.907	0.431
HBS			4.392				0.111
no problem	2.000	1.138	3.089	7.386	0.794	68.679	0.079
almost no problem	0.735	0.961	0.585	2.086	0.317	13.730	0.444
LBS			1.479				0.477
almost no problem	− 0.968	0.809	1.431	0.380	0.078	1.855	0.232
low-level problem	− 0.562	0.669	0.705	0.570	0.153	2.117	0.401
DQD			1.624				0.444
almost no problem	− 1.028	1.702	0.365	0.358	0.013	10.049	0.546
low-level problem	− 1.464	1.610	0.827	0.231	0.010	5.428	0.363
Tobacco use			0.113				0.945
Never smokers	0.139	0.465	0.089	1.149	0.462	2.860	0.765
Former smokers	0.030	0.532	0.003	1.030	0.363	2.921	0.955
Alcohol use			1.343				0.511
Never drinkers	− 0.445	0.434	1.051	0.641	0.273	1.501	0.305
Former drinkers	− 0.579	0.586	0.979	0.560	0.178	1.765	0.322
Constant	2.897	1.915	2.288	18.125			0.130

Significant are in value [bold].

*PSQI* pittsburgh sleep quality index; *LBS* the lower bound score; *HBS* the higher bound score; *DQD* the diet quality distance; *CI* confidence interval; *OR* odds ratio.

## Discussion

The purpose of the study was to explore the association between ADL and daily healthy behaviors among older adults in China. The logistic regression analysis results showed that exercise and physical examination significantly associated ADL disability. The findings of this study support that healthy behaviors in daily living may be effective in promoting positive and healthy aging, especially among older adults.

We observed ADL in 706 older adults in Chengdu, Southwest China. A total of 169 of the 706 older adults (23.9%) were disabled in ADL. The present study revealed that older adults had a higher ADL disabled ratio, which was consistent with previous research, which reported that approximately 31.06% of elderly individuals have limited activities of daily living due to health reasons^32^. Qian’s study^33^ investigated B-ADL and I-ADL disability rates of elderly individuals over 60 years old in China, which were 23.8 and 35.4%, respectively. In Hao’s study^34^, they investigated 1083 residents (≥ 60 years old) and found that 15.4% of them were partially disabled and 16.6% of them were fully disabled. In contrast, the proportion of ADL impairment was lower in studies with an American population (18%) in a study by Banks^35^. The differences between our study and Banks’s study may be due to the type of race.

Our logistic regression analysis showed that age, resident, chronic disease, exercise and physical examination were significantly correlated with ADL in older adults, which was consistent with the study by Qiao^36^. In Qiao’s study^36^, they used data from the China Health and Retirement Longitudinal Study (CHARLS) and the Survey of Health, Ageing and Retirement in Europe (SHARE) and supported a strong bidirectional association between disability and multimorbidity among middle-aged and elderly adults. In another study by Chen^37^, the factors that predicted disability were gender, age, educational level, the number of chronic diseases, and whether someone had metabolic syndrome.

However, there were some different findings between our study and others. In Alfonso’s study, they found that among a total of 943 participants from the FRADEA Study with a higher number of diseases, 14 preselected diseases were not associated with incident disability in ADL in Spanish^38^. Ćwirlej-Sozańska’s study involved 426 subjects aged 71–80 years and reported that the strongest factors related to difficulties with ADL were assessment of satisfaction with life, using assistive devices, and having one's home suitably adapted^39^. These differences are likely due to the populations, cultures and regions differing across studies.

Another important finding from our study is that exercise was significantly correlated with ADL in older adults, which was consistent with several studies. An included meta-analysis of (prospective) longitudinal studies for the prevention of onset and progression of basic activities of daily living (BADL) disability by physical activity 22 concluded that there was a 49% reduction in the incidence of BADL disability in older adults (aged ≥ 50 years) with a medium/high level of physical activity compared with those with a low physical activity level^40^. A medium/high physical activity level vs low levels of physical activity also reduced the progression of BADL disability by 45%^41^. The preventative effect was found in both older (≥ 75 years) and younger (< 74 years) individuals with and without diseases and in older adults who already had functional limitations or disability^40^. Other random effects meta-analyses revealed significant, beneficial effects of physical activity on ADL physical performance, with the largest effects found for moderate physical activity levels and for activity types with high levels of mental (e.g., memory, attention), physical (e.g., coordination, balance) and social (e.g., social interaction) demands^42^.

Periodic physical examination contributes to early detection and timely treatment and is considered helpful in preventing illness and promoting health among elderly individuals. In some situations, such as screening for cancer, periodic physical examination is very important^43^. Few studies have focused on the relationship between physical examination and ADL in older adults. We found that individuals who have an annual physical examination would have better ADL in daily life. Kazuyoshi’s study^44^ found that bone mineral density, grip strength, 10-m gait time, back muscle strength, and two-step test were all significantly lower in the group with a lower frequency of participation in the checkup. The government and public health institutions should give special attention to older adults and help them to acquire the habit of having an annual physical examination.

### Strengths and limitations

The findings of the current study are expected to provide valuable evidence for community nurses to identify the relationships for the ADL-disabled in healthy behaviors in an effort to extend health expectancy. This is the first observational study investigating healthy behaviors, including tobacco use, alcohol use, exercise, dietary behavior, sleep behavior and physical examination and ADL, in older adults in China. Second, we had a large sample including 706 older adults in Southwest China. Third, our group used 2 trained research nurses to reduce differences stemming from subjective observation.

However, there are several limitations that must be discussed. First, the cross-sectional study limits the causality, and a bidirectional relationship between healthy factors and ADL would be considered as well. Second, because the study was conducted in a single city in China, the results are not considered representative of other districts in China. Considering these limitations, further studies are warranted to verify these findings.

## Conclusions

This study showed that older adults had a higher ADL disabled ratio, and 169 of the 706 older adults (23.9%) were disabled in ADL. In addition, we explored the risk factors related to ADL disability and found that age, resident, chronic disease, exercise and physical examination were associated with ADL among older adults. The study indicat that medium/high exercise may be a protective factor for older adults, and community nurses can encourage older adults to exercise when carrying out primary prevention measures. The government and public health institutions should give special attention to older adults and help them to acquire the habit of having an annual physical examination.

## Data Availability

Data are available upon reasonable request to the first author.
