# Publisher Correction: Association of daily health behavior and activity of daily living in older adults in China

**DOI:** 10.1038/s41598-023-48289-w

**Published:** 2023-11-28

**Authors:** Ling-Ying Wang, Mei Feng, Xiu-Ying Hu, Meng-Lin Tang

**Affiliations:** 1 West China Hospital Critical Care Medicine Department, Sichuan University/West China School of Nursing, Sichuan University, Chengdu, 610041 China; 2 Innovation Center of Nursing Research and Nursing Key Laboratory of Sichuan Province, West China Hospital, Sichuan University/West China School of Nursing, Sichuan University, Chengdu, 610041 China

Correction to: *Scientific Reports* https://doi.org/10.1038/s41598-023-44898-7, published online 09 November 2023

In the original version of this Article, Xiu-ying Hu was omitted as a corresponding author. Correspondence and requests for materials should also be addressed to xiuyinghu@scu.edu.cn.

The original Article has been corrected.

